# Problematic Social Media Use in Sexual and Gender Minority Young Adults: Observational Study

**DOI:** 10.2196/23688

**Published:** 2021-05-28

**Authors:** Erin A Vogel, Danielle E Ramo, Judith J Prochaska, Meredith C Meacham, John F Layton, Gary L Humfleet

**Affiliations:** 1 Stanford Prevention Research Center Department of Medicine Stanford University Stanford, CA United States; 2 Hopelab San Francisco, CA United States; 3 Department of Psychiatry and Behavioral Sciences University of California San Francisco, CA United States

**Keywords:** sexual and gender minorities, social media, Facebook, internet, social stigma, mobile phone

## Abstract

**Background:**

Sexual and gender minority (SGM) individuals experience minority stress, especially when they lack social support. SGM young adults may turn to social media in search of a supportive community; however, social media use can become problematic when it interferes with functioning. Problematic social media use may be associated with experiences of minority stress among SGM young adults.

**Objective:**

The objective of this study is to examine the associations among social media use, SGM-related internalized stigma, emotional social support, and depressive symptoms in SGM young adults.

**Methods:**

Participants were SGM young adults who were regular (≥4 days per week) social media users (N=302) and had enrolled in Facebook smoking cessation interventions. As part of a baseline assessment, participants self-reported problematic social media use (characterized by salience, tolerance, and withdrawal-like experiences; adapted from the Facebook Addiction Scale), hours of social media use per week, internalized SGM stigma, perceived emotional social support, and depressive symptoms. Pearson correlations tested bivariate associations among problematic social media use, hours of social media use, internalized SGM stigma, perceived emotional social support, and depressive symptoms. Multiple linear regression examined the associations between the aforementioned variables and problematic social media use and was adjusted for gender identity.

**Results:**

A total of 302 SGM young adults were included in the analyses (assigned female at birth: 218/302, 72.2%; non-Hispanic White: 188/302, 62.3%; age: mean 21.9 years, SD 2.2 years). The sexual identity composition of the sample was 59.3% (179/302) bisexual and/or pansexual, 17.2% (52/302) gay, 16.9% (51/302) lesbian, and 6.6% (20/302) other. The gender identity composition of the sample was 61.3% (185/302) cisgender; 24.2% (73/302) genderqueer, fluid, nonbinary, or other; and 14.6% (44/302) transgender. Problematic social media use averaged 2.53 (SD 0.94) on a 5-point scale, with a median of 17 hours of social media use per week (approximately 2.5 h per day). Participants with greater problematic social media use had greater internalized SGM stigma (*r*=0.22; *P*<.001) and depressive symptoms (*r*=0.22; *P*<.001) and lower perceived emotional social support (*r*=−0.15; *P*=.007). Greater internalized SGM stigma remained was significantly associated with greater problematic social media use after accounting for the time spent on social media and other correlates (*P*<.001). In addition, participants with greater depressive symptoms had marginally greater problematic social media use (*P*=.05). In sum, signs of problematic social media use were more likely to occur among SGM young adults who had internalized SGM stigma and depressive symptoms.

**Conclusions:**

Taken together, problematic social media use among SGM young adults was associated with negative psychological experiences, including internalized stigma, low social support, and depressive symptoms. SGM young adults experiencing minority stress may be at risk for problematic social media use.

## Introduction

### Background

Despite sweeping social change in many parts of the United States, many sexual and gender minority (SGM) youth and young adults face stigma, prejudice, and discrimination [1]. With social media’s broad reach and its potential for supportive interactions, SGM individuals—especially SGM youth and young adults—may seek connection, validation, and community-building on social media. Indeed, SGM individuals use social media more frequently than the average US adult [2]. However, social media use can become problematic by interfering with functioning in daily life [3]. SGM young adults experiencing processes and effects of minority stress (ie, internalized stigma, depressive symptoms, and low emotional social support) may also be at risk for problematic social media use.

As a first step toward informing recommendations for health social media use for SGM young adults, it is important to identify those who may be at risk for problematic social media use (eg, preoccupation with social media use and difficulty with reducing or abstaining from use). In a sample of SGM young adults participating in clinical trials of 2 Facebook-delivered interventions for smoking cessation, this study examined the associations between problematic social media use and internalized SGM stigma, emotional social support, and depressive symptoms. We first review problematic social media use, including its theoretical underpinnings and correlates, among young adults. We then review the minority stress experienced by SGM young adults, social media use among SGM young adults, and how minority stress may influence the development of problematic social media use. Finally, we present potential correlates of problematic social media use in SGM young adults, informed by theoretical models of problematic social media use and minority stress.

### Problematic Social Media Use

Social media use is almost ubiquitous among young adults, with 91% of young adults in the United States reporting the use of at least one social media platform in a 2019 survey [4]. In some social media users, habitual use leads to problematic use, characterized by interference with functioning, negative feelings when unable to access social media, inability to control one’s social media use, and dominance of social media use over other thoughts and behaviors [5]. Young adults are particularly vulnerable to problematic social media use compared with older adults [5]. Numerous studies have examined the predisposing risk factors for problematic social media use among young adults. According to the Interaction of Person-Affect-Cognition-Execution (I-PACE) model, problematic social media use occurs when individuals with predisposing risk factors experience gratifications (eg, stress relief, positive mood) from social media use [6]. Such gratifications reinforce the use of social media, which can then escalate until it becomes problematic (ie, becomes difficult to control and creates negative consequences) [6].

In the general young adult population, predisposing risk factors associated with vulnerability to problematic social media use include certain personality traits, mental health symptoms, and experiences. First, high extraversion [3,7,8] and low conscientiousness [3,7-9] are consistently associated with problematic social media use across studies. Social media offers an outlet for connecting with others in a way that requires little planning and can be an escape from other daily tasks [7]. Second, young adults who experience depressive symptoms are at risk of problematic social media use. A nationally representative study of young adults in the United States found that problematic social media use was associated with a 9% increase in the odds of experiencing depressive symptoms [10]. Individuals with depressive symptoms may use social media to relieve stress and seek social support, thereby developing problematic social media use patterns [11]. Third, young adults who frequently experience fear of missing out (FOMO) are more likely to exhibit problematic social media use [8,12-14]. FOMO refers to an uncomfortable feeling that others are having positive experiences in one’s absence and is associated with higher engagement in social media use [15]. In line with the I-PACE model, these factors may interact to influence problematic social media use. For example, depressive symptoms may interfere with an individual’s ability to socialize and enjoy experiences, thereby producing FOMO. Consistent with the I-PACE model, a three-wave longitudinal study found that Chinese young adults who experienced greater depressive symptoms subsequently experienced more FOMO, followed by more problematic smartphone use [16]. In sum, predisposing factors to problematic social media use in the general young adult population include personality traits, mental health concerns, and social cognition.

### Minority Stress Among SGM Young Adults

SGM young adults face unique challenges and may have different predispositions than their non-SGM peers because of their experiences of minority stress. The minority stress model, as applied to sexual and gender minorities, posits that being a minority leads to chronic experiences of stigma, prejudice, and discrimination as well as more proximal stress processes, including vigilance toward future rejection, the concealment of one’s sexual or gender identity, and the internalization of negative societal attitudes toward SGM individuals (ie, internalized stigma) [17]. Both proximal and distal stress processes can lead to negative mental health outcomes, including depressive symptoms [17-19]. Receiving adequate social support may ameliorate the negative effects of minority stress on SGM youth and young adults’ depressive symptoms [20]. In other words, SGM individuals who experience negative effects of minority stress may report internalized SGM stigma, depressive symptoms, and low emotional social support. These processes and outcomes of minority stress may contribute to problematic social media use, especially if SGM individuals turn to social media for social support but still feel unsupported.

### Social Media Use Among Sexual and Gender Minorities

Social media may help undersupported individuals compensate for their lack of in-person emotional social support [21]. In a qualitative study, transgender adolescents reported using social media to obtain emotional support and inspiration from other transgender individuals and to obtain information about gender-affirming therapy [22]. Alternatively, social media use may have an overall negative effect on the mental health of SGM young adults. Transgender adolescents also report seeing hurtful content related to their gender identity [22]. SGM social media users face the unique challenge of managing disclosure of their SGM identity on social media, where different members of their social networks (eg, friends, family, and coworkers) may be able to see their posts [23]. A systematic review of the association between social media use and depressive symptoms among SGM individuals concluded that social media had both positive effects (eg, connection with the SGM community and self-expression) and negative effects (eg, being a victim of cyberbullying and experiencing the stress of hiding one’s SGM identity from certain others) [24]. A study conducted in China found that among SGM individuals aged 13-58 years, problematic social media use was associated with greater depressive symptoms, time spent on social media, and web-based social support seeking [25]. However, these specific relationships have not yet been studied among young adults in the United States, and there may be important cross-cultural differences in social media use and in the social experience of identifying as SGM. In addition, Han et al [25] did not measure offline emotional social support; therefore, it is unclear whether SGM individuals who lacked offline emotional social support engaged in more problematic social media use.

### This Research and Hypotheses

SGM young adults are vulnerable to internalized stigma, low emotional social support, and depressive symptoms as part of minority stress. The I-PACE model suggests that these predispositions may lead to problematic social media use in response to stress. Although research has examined predisposing factors for problematic social media use in the general young adult population, the effects and processes of minority stress as potential predispositions for problematic social media use in SGM young adults have not been explored.

This study is largely exploratory, testing the associations between problematic social media use and individual differences (informed by the I-PACE and minority stress models) in a convenience sample of SGM young adults enrolled in clinical trials of 2 smoking cessation interventions delivered on Facebook. Nonetheless, the extant literature and the minority stress model provided a framework for testing relevant factors that may predispose SGM young adults to problematic social media use. Specifically, we predicted that problematic social media use would be associated with greater internalized SGM stigma, lesser emotional social support, and greater depressive symptoms. Problematic social media use was expected to be positively associated, but not synonymous, with the time spent on social media [25-27]. Overall, this study assessed the relative strength of the associations among problematic social media use, time spent on social media, internalized SGM stigma, social support, and depressive symptoms, in a cross-sectional study of SGM young adults enrolled in 2 smoking cessation intervention trials on Facebook.

## Methods

### Participants and Procedures

Data were taken from the baseline assessments of 2 randomized controlled trials that tested the efficacy of SGM-tailored Facebook smoking cessation interventions compared with similar, nontailored interventions. Participants (N=302) were aged between 18 and 25 years, identifying as SGM (ie, nonheterosexual and/or noncisgender), having smoked at least 100 cigarettes in their lifetime, using Facebook for at least 4 days per week, living in the United States, and were well-versed in English. Participants in both trials were recruited between April and December 2018 with an SGM-tailored Facebook advertising campaign [28]. Clicking on an ad directed the participants to a confidential screener. After verifying their age and identity, eligible participants received a baseline web-based survey. Relevant measures from the baseline web-based survey are described in the *Measures* section. Informed consent was obtained from all participants, and all research activities were approved by the University of California, San Francisco, Institutional Review Board.

### Measures

#### Problematic Social Media Use

Problematic social media use was measured using the Bergen Social Media Addiction Scale [29]. Participants rated, on a 1-5 Likert-type scale, the degree to which 6 statements about their social media use were true for them (1=very rarely; 2=rarely; 3=sometimes; 4=often; 5=very often). The possible range of scores was 1-5. Sample items included “You spend a lot of time thinking about social media or planning how to use it” and “You become restless or troubled if you are prohibited from using social media.”

#### Time Spent on Social Media

Participants answered, “Approximately how many hours per week do you spend using social media?” The item was a free response; the natural log of responses was used in analyses.

#### Frequency of Social Media Use

A total of 9 items assessed the frequency of use of 8 social media platforms (Facebook, Instagram, Snapchat, Twitter, Pinterest, LinkedIn, Reddit, and Tumblr), plus a write-in “other” option, using 1-5 Likert-type scales (1=never; 2=monthly; 3=weekly; 4=once a day; 5=multiple times a day). The possible scores for each platform ranged from 1-5.

#### Internalized SGM Stigma

To measure internalized SGM stigma, participants completed the Revised Internalized Homophobia Scale, a 5-item measure adapted for all SGM identities [30] by rating their agreement with 5 statements on a 1-5 Likert-type scale (1=strongly disagree; 2=disagree; 3=neutral; 4=agree; 5=strongly agree), with possible average scores ranging from 1-5. Sample items included “I wish I weren’t LGBTQ+ (lesbian, gay, bisexual, transgender, queer+)” and “I have tried to stop being LGBTQ+.”

#### Perceived Emotional Social Support

The 8-item Emotional Support subscale of the National Institutes of Health (NIH) Toolbox for the assessment of neurological and behavioral functions measured the perceived emotional social support [31]. Sample items include, “I have someone who understands my problems” and “I feel there are people I can talk to if I am upset” rated on a 1-5 Likert-type scale (1=never; 5=always), with total scores ranging from 8-40.

#### Depressive Symptoms

Depressive symptoms were measured using the 2-item Patient Health Questionnaire, a validated screener assessing the frequency of depressed mood and anhedonia in the past 2 weeks, with possible scores ranging from 0-6 [32]. The 2-item Patient Health Questionnaire is sensitive to changes in depressive symptoms and has been found to detect symptom improvement during depression treatment [33].

#### Demographic Characteristics

The participants reported their age and sex assigned at birth (male or female). Participants selected all applicable terms to describe their gender identity (male/man, female/woman, trans male/trans man, trans female/trans woman, genderqueer/gender nonconforming, genderfluid, agender, nonbinary, or different identity), sexual identity (straight [heterosexual], lesbian/gay [homosexual], bisexual, pansexual, or not listed), and race and ethnicity (American Indian/Alaska Native, Asian, Black, Hispanic, Pacific Islander/Native Hawaiian, White, or other). Household income was presented in 7 ranges from “less than US $20,000” to “over US $200,000.” Subjective social status was measured on a 1-10 scale from “worst off” to “best off” compared with others in the United States and in their communities [34]. Years of education were measured with “How many total years of school have you completed?”

### Statistical Analysis

The analyses were conducted in 3 steps. First, we examined the distributions and reliability of the measures before creating composite scores. Second, we examined bivariate Pearson correlations between problematic social media use, social media use in hours per week, internalized SGM stigma, social support, and depressive symptoms. Third, significant correlates of problematic social media use identified in bivariate screening were entered into a multiple linear regression analysis as independent variables, with problematic social media use as the outcome dependent variable. As gender identity was significantly associated with problematic social media use, we adjusted for gender identity by entering it in step 1. The main correlates of interest were entered as independent variables in step 2. In the final model, gender identity (control variable) was entered in step 1; time spent on social media, internalized SGM stigma, emotional social support, and depressive symptoms (independent variables) were entered in step 2, and problematic social media use was entered as the dependent variable.

## Results

### Sample Characteristics

The sample characteristics are listed in Table 1. Most participants (218/302, 72.2%) were assigned female at birth; however, they varied in their current gender identity and sexual identity. The sample comprised 61.3% (185/302) cisgender participants; 24.2% (73/302) genderqueer, fluid, and/or nonbinary participants; and 14.6% (44/302) transgender participants. Most participants were bisexual and/or pansexual (179/302, 59.3%), followed by gay (52/302, 17.2%), lesbian (51/302, 16.9%), or other (20/302, 6.6%) participants. The sample majority (188/302, 62.3%) was non-Hispanic White. Participants perceived themselves as below average in social standing compared with others in the United States (mean 3.91, SD 1.61) and their communities (mean 4.32, SD 1.96), where the scales ranged from 1-10.

**Table 1:** Participant characteristics (N=302).

Characteristics		Value
Age (years), mean (SD)		21.9 (2.2)
Sex assigned at birth (female), n%		218 (72.2)
**Gender identity^a^, n (%)**		
	Cisgender	185 (61.3)
	Transgender	44 (14.6)
	Genderqueer, fluid, and/or nonbinary	73 (24.2)
**Sexual identity^b^, n (%)**		
	Gay	52 (17.2)
	Lesbian	51 (16.9)
	Bisexual/pansexual	179 (59.3)
	Other	20 (6.6)
**Race and ethnicity, n (%)**		
	Non-Hispanic White	188 (62.3)
	Native American/Alaskan Native	3 (1)
	African American/Black	3 (1)
	Asian	10 (3.3)
	Hispanic/Latinx	29 (9.6)
	Pacific Islander/Hawaiian Native	2 (0.7)
	More than one race and ethnicity	59 (19.5)
	Other	8 (2.6)
**Household income, n (%)**		
	Less than US $20,000	75 (24.8)
	US $21,000-US $60,000	155 (51.3)
	US $61,000-US $100,000	52 (17.2)
	More than US $100,000	20 (6.6)
**Subjective social status (out of 10), mean (SD)**		
	Compared with others in the United States	3.9 (1.6)
	Compared with others in your community	4.3 (2.0)
Years of education, mean (SD)		13.5 (2.1)

^a^Participants selected all applicable terms from straight (heterosexual), lesbian/gay (homosexual), bisexual, pansexual, and not listed (please specify). Sexual identity was recoded into the categories presented here. When multiple identities were selected, “Gay/lesbian” took priority in coding, followed by “bisexual/pansexual” then “other” [28].

^b^Participants selected all applicable terms from male/man, female/woman, trans male/trans man, trans female/trans woman, genderqueer/gender nonconforming, genderfluid, agender, nonbinary, or different identity (please specify). Gender identity was recoded into the categories presented here. When multiple identities were selected, “transgender” took priority, followed by “cisgender” then “other” [28].

### Distributions of Measures

The distributions of the key measures are presented in Table 2. Composite scores were computed using the mean of the internalized homophobia items (α=.74), sum of social support items (α=.96), sum of depressive symptoms items (*r*=0.75), and mean of problematic social media use items (α=.84), according to the recommended scoring guidelines for each measure. Owing to a skewed distribution, the natural log of social media use frequency was used in the analyses.

**Table 2:** Distributions of key measures.

Measure		Respondents, n (%)	Mean (SD) or median (IQR)	Observed range	Possible range	Scale	
Problematic social media use (score)		302 (100)	2.5 (0.9)	1-5	1-5	1=very rarely; 2=rarely; 3=sometimes; 4=often; 5=very often	
Social media (hours per week)		300 (99.3)	17.0 (10-30)	1-120	≥0	Measured continuously	
Natural log (Ln) of hours per week		300 (99.3)	2.8 (0.7)	0.7-4.8	≥0	Measured continuously	
**Frequency of platform use (score)**							1=never; 2=monthly; 3=weekly; 4=once/day; 5=multiple times/day
	Facebook	302 (100)	4.7 (0.6)	2-5	1-5		
	Instagram	301 (99.7)	4.0 (1.3)	1-5	1-5		
	Snapchat	300 (99.3)	3.7 (1.4)	1-5	1-5		
	Twitter	297 (98.3)	2.5 (1.6)	1-5	1-5		
	Pinterest	292 (96.7)	1.8 (1.1)	1-5	1-5		
	LinkedIn	293 (97)	1.3 (0.7)	1-4	1-5		
	Reddit	292 (96.7)	1.9 (1.3)	1-5	1-5		
	Tumblr	297 (98.3)	2.6 (1.5)	1-5	1-5		
	Other	237 (78.5)	1.2 (0.7)	1-5	1-5		
Internalized SGM^a^ stigma (score)		299 (99)	1.7 (0.8)	1-5	1-5	1=strongly disagree; 2=disagree; 3=neutral; 4=agree; 5=strongly agree	
Emotional social support (score)		302 (100)	30.7 (6.9)	11-40	8-40	1=never; 2=rarely; 3=sometimes; 4=usually; 5=always (sum of 8 items)	
Depressive symptoms (score)		302 (100)	3.5 (1.8)	0-6	0-6	0=not at all and 3=nearly every day (sum of 2 items)	

^a^SGM: sexual and gender minority.

On average, problematic social media use was around the midpoint of the 1-5 scale (mean 2.5, SD 0.9). Median use of social media was 17 hours per week (an average of approximately 2.5 h per day). The 3 most frequently used social media platforms were Facebook, Instagram, and Snapchat. Internalized SGM stigma scores, which can range from 1-5, averaged 1.7 (SD 0.8), which was comparable with previous research (mean 1.5, SD 0.7 [30]). Perceived emotional social support was moderate (mean 30.7, SD 6.9), on a scale from 8-40; depressive symptoms averaged 3.5 (SD 1.8) on a scale from 0-6.

### Bivariate Correlates of Problematic Social Media Use

Problematic social media use was significantly greater among participants who spent more time on social media (*r*=0.24; *P*<.001)*.* Participants with greater problematic social media use also reported greater internalized stigma about their SGM identities (*r*=0.22; *P*<.001), perceived less emotional social support (*r*=−0.16; *P*=.007), and had greater depressive symptoms (*r*=0.22; *P*<.001) compared with those with lower problematic social media use. Participants who spent more time on social media had significantly greater depressive symptoms (*r*=0.15; *P*=.009); however, time spent on social media was not associated with internalized SGM stigma (*r*=−0.05; *P*=.41) or emotional social support (*r*=0.02; *P*=.78). The correlations between all of the measures are presented in Table 3.

**Table 3:** Pearson correlations between key variables (N=302)^a^.

Measure			Problematic social media use		Social media hours per week (natural log [Ln])		Internalized sexual and gender minority stigma		Emotional social support		Depressive symptoms
**Problematic social media use**											
	*r*	1		0.24^b^		0.22^b^		−0.16^c^		0.22^b^	
	*P* value	—^d^		<.001		<.001		.007		<.001	
**Social media hours per week (natural log [Ln])**											
	*r*	0.24^b^		1		−0.05		0.02		0.15^c^	
	*P* value	<.001		—		.41		.78		.009	
**Internalized sexual and gender minority stigma**											
	*r*	0.22^b^		−0.05		1		−0.23^b^		0.13^e^	
	*P* value	<.001		.41		—		<.001		.02	
**Emotional social support**											
	*r*	−0.16^c^		0.02		−0.23^b^		1		−0.28^b^	
	*P* value	.007		.78		<.001		—		<.001	
**Depressive symptoms**											
	*r*	−0.22^b^		0.15^c^		0.13^e^		−0.28^b^		1	
	*P* value	<.001		.009		.02		<.001		—	

^a^n=300 for social media hours per week; n=299 for internalized sexual and gender minority stigma.

^b^Significant at the *P*<.001 level.

^c^Significant at the *P*<.01 level.

^d^Not applicable.

^e^Significant at the *P*<.05 level.

### Multivariable Model of Problematic Social Media Use

Time spent on social media (β=.24; *P*<.001) and internalized SGM stigma (β=.20; *P*<.001) remained significantly and positively associated with problematic social media use in the adjusted multiple linear regression analysis (presented in Table 4). The positive association between depressive symptoms and problematic social media use was marginally significant in the adjusted multiple linear regression analysis (β=.11; *P*=.05).

**Table 4:** Adjusted multiple linear regression analysis with problematic social media use as the outcome variable.

Variable				β		*P* value
**Step 1**						
	**Gender identity**					
		Transgender (reference: cisgender)	.04		.48	
		Nonbinary and/or other gender (reference: cisgender)	.18		.002	
**Step 2**						
	**Gender identity**					
		Transgender (reference: cisgender)	.02		.66	
		Nonbinary and/or other gender (reference: cisgender)	.15		.009	
	Social media (hours per week; natural log [Ln])			.24		<.001
	Internalized stigma			.20		<.001
	Emotional social support			−.06		.28
	Depressive symptoms			.11		.05

## Discussion

### Principal Findings

In this study of SGM young adults enrolled in 2 Facebook smoking cessation intervention trials, those with greater problematic social media use had greater internalized SGM stigma and depressive symptoms and lower perceived emotional social support. Greater internalized SGM stigma remained significantly associated with greater problematic social media use after accounting for the time spent on social media and other correlates. In addition, participants with greater depressive symptoms had marginally greater problematic social media use. Overall, signs of problematic social media use were more likely to occur among SGM young adults who had internalized SGM stigma and depressive symptoms.

### Comparison With Prior Work

These results are consistent with our hypothesis that SGM young adults with greater internalized SGM stigma would be more likely to exhibit problematic social media use than those with lower internalized SGM stigma. According to the I-PACE model, personal characteristics can predispose individuals to problematic social media use. Internalized SGM stigma may be one such characteristic for SGM young adults. Individuals with poor self-concepts often seek social support on social media, and these attempts are often unsuccessful. Specifically, previous research found that adults with low self-esteem primarily posted negative thoughts [35] and sought reassurance [36]. Such posts are less likely to garner social support than positive posts [37]. Similarly, SGM young adults who feel marginalized and different from their peers may find that these feelings are exacerbated by the positive self-presentation bias on social media [38]. Seeing others appear to be happy and comfortable with themselves may be harmful to SGM young adults who internalize negative messages about their sexual and gender identities. Therefore, problematic social media use may contribute to further internalization of stigma. The I-PACE model posits bidirectional relationships between predispositions to problematic social media use and social media use behavior. Although this cross-sectional analysis cannot determine causality, the results are consistent with the I-PACE model and suggest that internalized SGM stigma may be a predisposing factor for problematic social media use.

SGM young adults with low emotional social support were more likely to have problematic social media use in bivariate analyses, thereby corroborating the associations between low social support and problematic social media use found in the general population [39] and partially supporting our hypothesis. The association did not remain significant in the multivariable model, suggesting that internalized SGM stigma accounted for more variance in problematic social media use than did emotional social support. SGM young adults who experienced lower emotional social support also reported greater internalized SGM stigma. According to the minority stress model, internalized stigma and low emotional social support can result from experiencing prejudice and discrimination [18]. Given the frequency with which SGM individuals experience prejudice and discrimination [1], assessing SGM individuals’ emotional support is critical. Low emotional social support may be an additional risk factor for problematic social media use, albeit a weaker predictor than internalized SGM stigma.

Problematic social media use was associated with depressive symptoms in bivariate analyses, partially supporting our hypothesis. This is consistent with the I-PACE model and with extant research examining the link between depressive symptoms and problematic social media use in the general population [11,40].

Consistent with extant literature involving young adult social media users [41,42] and SGM social media users in China [25], SGM young adults in this study who spent more time on social media experienced more depressive symptoms. Frequent social media use may lead to depressive symptoms through social mechanisms such as FOMO [43]. Experiencing depression may also increase the risk of frequent social media use, as browsing social media may provide a temporary escape that requires little energy [27]. SGM individuals are at a high risk of depression [17]. Problematic social media use should be assessed as both a contributing factor to an individual’s depressive symptoms and the result of depressive symptoms.

This pattern of results suggests multiple predisposing factors for problematic social media use among SGM young adults, especially internalized SGM stigma. Social media may appeal to young adults with poor self-concepts and insufficient emotional support, as social media presents opportunities to connect with others. However, social media may not be an adequate source of support and connection for SGM young adults with poor self-views. Our results suggest that SGM young adults who used social media intensively, to the point of problematic use, still had high internalized SGM stigma, high depressive symptoms, and low emotional support. Moreover, SGM young adults with problematic social media use may withdraw from offline social opportunities and thus receive less support. Importantly, the relationship between problematic social media use and social support was significant only in bivariate analyses, suggesting that internalized SGM stigma is more strongly associated with problematic use. Problematic social media use was also associated with depressive symptoms in bivariate analyses. Seeking social support and not receiving it may exacerbate depressive symptoms [44].

Conversely, spending more time on social media was not associated with internalized stigma or social support. SGM young adults who experience distress around their identities may be more vulnerable to problematic social media use, regardless of how frequently they use social media. Consistent with previous research, time spent on social media and problematic social media use were modestly correlated but distinct [25-27]. Spending more time on social media did not appear to account for the relationships between problematic social media use, internalized SGM stigma, and depressive symptoms. SGM young adults who spent more time on social media but did not experience impairment or distress from their social media use may have used social media differently than those with greater problematic use. Problematic social media use may be a stronger indicator or consequence of underlying concerns (eg, internalized stigma and low social support) than the time spent on social media.

### Implications

This study supports and extends prior research on SGM individuals’ social media use by demonstrating associations between problematic use and common challenges faced by SGM young adults. When used in moderation, social media offers benefits such as self-expression and connection with others [24]. Although this study cannot confirm the direction of the associations among problematic social media use, internalized SGM stigma, low social support, and depressive symptoms, the results suggest that intense social media use may not meet the psychosocial needs of SGM young adults. Importantly, the time spent on social media was not significantly associated with internalized SGM stigma (*P*=.411) or low emotional social support (*P*=.775). SGM young adults with problematic social media use may represent a high-risk subset of social media users who are likely to experience social and mental health challenges. SGM young adults are vulnerable to problematic social media use, internalized stigma, low emotional support, and depressive symptoms. Clinicians working with SGM young adults should discuss social media use with clients and explore how social media may reflect or affect clients’ mental health. Social media may be an effective intervention platform for helping young adults improve their well-being and engage in health-promoting behaviors (eg, smoking cessation [45]).

### Limitations

First, this cross-sectional, correlational study design did not confirm causal inferences about the role of social media use in mental and physical health outcomes. Longitudinal data would enable the use of more sophisticated statistical modeling to understand the interplay among experiences of stigma, social support, and depressive symptoms. This study underscores the need for future research on the social media use of SGM young adults. Second, all participants were young adults who self-identified as SGM and smoked cigarettes. Other dimensions of sexual orientation (ie, sexual attraction and sexual behavior) were not assessed. SGM young adults who smoke may be a particularly vulnerable subset of SGM young adults, and it is unclear whether the results can be generalized to SGM individuals who do not smoke. Future research should aim to oversample non-White young adults and those assigned male at birth, as the sample in this study was mainly White (188/302, 62.3%) and assigned female at birth (218/302, 72.2%). Third, all participants were frequent Facebook users. Future research could aim to recruit SGM young adults from other social media platforms. Finally, this study was a secondary analysis that captured only a few potential correlates of problematic social media use. Measures of some constructs (eg, depressive symptoms and time spent on social media) were brief mental health symptoms aside from depression (eg, anxiety symptoms) were not measured, and “social media” was not defined. Future research could use more comprehensive measures to further our understanding of problematic social media use among SGM young adults.

### Conclusions

Although social media use can be beneficial for SGM young adults [24], it may not be an adequate source of social support and can become problematic. Problematic social media use is distinct from the time spent on social media and involves feelings of distress when disconnected from social media [5]. SGM young adults experiencing minority stress may be at risk for problematic social media use. Taken together, these results suggest that problematic social media use among SGM young adults is associated with internalized stigma, low social support, and depressive symptoms.

## Abbreviations

FOMO: fear of missing out
I-PACE: Interaction of Person-Affect-Cognition-Execution
LGBTQ+: lesbian, gay, bisexual, transgender, queer+
NIH: National Institutes of Health
SGM: sexual and gender minority
